# Metabolic Profiling and Cold-Starvation Stress Response of Oxygen-Tolerant *Lactobacillus gasseri* Strains Cultured in Batch Bioreactor

**DOI:** 10.3390/microorganisms7070200

**Published:** 2019-07-15

**Authors:** Diamante Maresca, Francesca De Filippis, Alessandro Robertiello, Gianluigi Mauriello

**Affiliations:** Department of Agricultural Sciences, University of Naples Federico II, 80055 Portici, NA, Italy

**Keywords:** aerobic and respiratory metabolism, lactic acid bacteria, cold-starvation stress, tricarboxylic acid cycle, in silico analysis

## Abstract

Phenotypic and genotypic evidence indicates that many LAB strains can grow in presence of oxygen and can shift from fermentative to aerobic and/or respiratory metabolism. The aerobic and respiratory growth of several LAB species have been studied, allowing the selection of strains showing improved biomass production, long-term survival, and resistance under oxygen and stress conditions. The aim of this work was to observe the adaptation of two *Lactobacillus gasseri* strains, described in a previous work, to aerobic (air injection) and respiratory (air injection plus hemin and menaquionone) conditions obtained in a batch bioreactor. One strain showed the higher biomass production and oxygen consumption as well as the lower acidification in respiratory condition. Instead, the other one grew better in aerobic condition, even though the higher resistance to cold-starvation stress was registered in respiratory condition. In silico analysis revealed notable differences between AL3 and AL5 genomes and that of the type strain. This work contributes to understanding the adaptation response of lactobacilli to aerobic and respiratory metabolism. We demonstrated that the supposed activation of respiratory metabolism may provide several modifications to cell physiology. These features may be relevant in some technological and health-promoting applications, including starter and probiotic formulations.

## 1. Introduction

The fermentative metabolism of lactic acid bacteria (LAB) has been intensively studied because of its technological implications in the food industry. In recent years, phenotypic and genotypic evidence has indicated that many LAB strains can grow in the presence of oxygen and can shift from fermentative to aerobic and/or respiratory metabolism [1]. In particular, some LAB strains can consume oxygen through the action of flavoprotein oxidases (e.g., NADH oxidase, NOX; pyruvate oxidase, POX; lactate oxidase, LOX; α-glycerophosphate oxidase) and can activate aerobic metabolism by the pyruvate oxidase-acetate/kinase (POX-ACK) pathway [2]. Moreover, several LAB strains can perform respiratory metabolism in the presence of oxygen by activating a minimal electron transport chain when exogenous heme and/or menaquinone are provided [3].

Aerobic and respiratory metabolism have been widely investigated in some strains of *Lactobacillus reuteri* and *Lactobacillus spicheri* [4], in the *Lactobacillus casei* group [5,6], and in the *Lactobacillus plantarum* group [7,8,9,10]. Moreover, some of these studies led to the selection of strains that improved the features of foods when used like adjunct cultures [11,12]. Advances in this area have led to an increased interest in the physiology, metabolism, and genetics of LAB’s aerobic lifestyle. However, very limited data are currently available on *Lactobacillus johnsonii* and *Lactobacillus gasseri*, although several strains have been extensively studied for their technological and probiotic properties [13,14]. *Lb. johnsonii* and *Lb. gasseri* belong to the *Lactobacillus acidophilus* group that was recently merged with the *Lactobacillus delbrueckii* group [15]. Currently, only the probiotic *Lb. johnsonii* NCC 533 has been studied to help provide a more global understanding of the molecular responses to the presence of oxygen [16,17].

Although genetic studies have helped explain the consequences of O_2_ and CO_2_ exposure on the physiology of *Lb. johnsonii* NCC 533, they have not fully addressed the complexity of the mechanisms of aerobic metabolism and the oxidative stress response. In our previous microplate screening study, the adaptation response to aerobic and respiratory cultivation was investigated in 34 *Lb. johnsonii/gasseri* strains isolated from the stools of breast fed babies [18]. The strains AL3 and AL5 were selected for their ability to grow under aerobic and/or respiratory conditions, as well as to scavenge hydrogen peroxide (H_2_O_2_) and/or reactive oxygen species (ROS) [18]. The whole-genome sequencing of both strains revealed that AL3 and AL5 belong to the *Lb. gasseri* species and that they possess genes involved in aerobic and respiratory metabolism and oxidative stress resistance [19].

The present work aimed to collect biochemical evidence (i.e., sugar consumption and metabolite production) of the activation of the aerobic and/or respiratory pathway in the strain *Lb. gasseri* AL3 and AL5 during their cultivation in a batch bioreactor. Furthermore, resistance to the cold-starvation stress of aerobic and respiratory cultures was assessed. Finally, a comparative in silico analysis was carried out to reveal differences in genome composition among AL3, AL5 and the type strain *Lb. gasseri* DSM 20243^T^.

## 2. Materials and Methods

### 2.1. Bacterial Strains

*Lactobacillus gasseri* AL3 and AL5 and *Lb. gasseri* DSM 20243^T^ were routinely propagated in aerobic condition at 37 °C for 24 h in Weissella Medium Broth pH 6.8 [9] modified by adding 10 g/L of fructose (mWMB).

### 2.2. Batch bioreactor cultivation

Strains were grown in mWMB at 37 °C for 30 h using a 1 L fermentation bioreactor (Applikon Biotechnology, Schiedam, Netherlands) under the following growth conditions: *i*) nitrogen (0.45 µm filter sterilised) flow at 0.1 vol/vol/min, stirrer speed 150 rpm (anaerobiosis, AN); *ii*) air (0.45 µm filter sterilised) flow at 0.1 vol/vol/min, stirrer speed 150 rpm (aerobiosis, AE); *iii*), AE condition supplemented with 2.5 µg/mL of hemin and 1 µg/mL of menaquinone (respiration, RS). The bioreactor was inoculated (2% vol/vol) with standardized (OD_650_ = 1.0) overnight pre-cultures obtained in static and normal atmospheric condition. Dissolved oxygen concentration (DO%) was measured using a polarographic electrode (Applikon Biotechnology, JG Delft, Netherland). Fermentation parameters such as DO%, pH, and temperature were monitored using the ezControl system (Applikon Biotechnology, JG Delft, Netherland). Foam formation was controlled by adding 0.5 mL/L of Antifoam 204 solution (Sigma-Aldrich, Saint Louis, MO, USA) at the medium composition. Three independent fermentation rounds were carried out for each experiment.

### 2.3. Bacterial Growth Monitoring

Bacterial growth was monitored by measuring the optical density of culture samples at 650 nm (OD_650_) using a spectrophotometer (Eppendorf BioSpectrometer, Milan, Italy). Samples were collected immediately after inoculation and then after 3, 4, 5, 6, 7, 8, 9, 20, 22, 24, 26, 28, and 30 h.

### 2.4. HPLC Analysis

Sugar consumption (glucose and fructose) and metabolite production (acetic, citric and lactic acid) were measured by HPLC analysis. Aliquots of 1 mL of AN, AE, and RS cultures was collected immediately after inoculation and then after 7, 9, 20, 22, 24, and 30 h of cultivation and centrifuged at 13,000 *g* for 5 min. The resulting supernatant was diluted in the mobile phase (H_2_SO_4_ 0.01 N) 1:5 (vol/vol) and filtered by AcroDisc (0.2 µm; Millipore, Burlington, MA, USA). Sugars and metabolites were quantified by a Gilson 307 Series HPLC system fitted with a MetaCarb 67H 6.5 × 300 mm column (Agilent Technologies, Santa Clara, CA, USA) in an oven at 65 °C. The column was eluted at 0.4 mL/min by a 1:9 (vol/vol) solution of H_2_SO_4_ in ultrapure water. A refractometer (RID 133, Gilson, Middleton, WI, USA) was used as a detector. Standards (47829 glucose, F2793 fructose, 46937 lactic acid, 71251 acetic acid and 46933 citric acid analytical standard, Sigma-Aldrich, St. Louis, MO, USA) were used for the quantification of different sugars in the samples.

### 2.5. Tolerance to Cold-Starvation Stress

Culture samples were collected at the early stationary phase, matching to 20 h in AE and 28 h in RS for AL3 and AL5, respectively, and 22 h in AN and AE conditions for *Lb. gasseri* 20243^T^. Samples were centrifuged at 6500 *g* for 10 min, washed twice in 20 mM potassium phosphate buffer pH 7.0 (PB7), and re-suspended in PB7 to obtain a final OD_650_ = 1. Tolerance to starvation was evaluated by storing cell suspensions at 4 °C and performing a viable count on MRS Agar (Oxoid) (37 °C in aerobiosis) at 0, 7, 14, 21, and 28 days of storage.

### 2.6. Comparative in Silico Analysis

*In silico* analysis was carried out in order to compare the presence of 33 genes (29 genes and *cyd* operon) coding for the main enzymes involved in aerobic (pyruvate oxidase (POX), lactate oxidase (LOX), L-aminoacid oxidase (LAO), NADH oxidase (NOX) and acetate kinase (ACK)), respiratory metabolism (NADH dehydrogenase (NDH), ubiquinone/menaquinone biosynthesis C-methylase (UbiE), cytochrome bd-I oxidase (CydABCD)) and oxidative stress response (NADH-peroxidase (NPR), glutathione reductase (GOR), glutathione peroxidase (GOP), γ-glutamylcystiene synthetase (GshA), glutathione synthetase (GshB), bifunctional glutamate-cysteine ligase/glutathione synthetase (GshF), thioredoxin reductase (TrxR), thioredoxin peroxidase (TrxP), superoxide dismutase (SOD), catalase (CAT), manganese catalase (Mn-CAT), catalase-peroxidase (CATG), and DNA binding protein from starved cells (Dps)) in finished genome of *Lb. gasseri* DSM 20243^T^ (NCBI database; http://www.ncbi.nlm.nih.gov) and in draft genome sequences of *Lb. gasseri* AL3 and AL5 (DDBJ/ENA/GenBank, accession numbers MTZT00000000 and MUJA00000000, respectively). Furthermore, predicted genes of *Lb. gasseri* AL3 and AL5 [19] were queried in the NCBI database against the main enzymes involved in the partial tricarboxylic acid (TCA) cycle of LAB, including gamma (CitD), beta (CitE), and alpha (CitF) subunits of citrate lyase (CL), citrate permease (CitP), oxaloacetate decarboxylase (AOD), pyruvate carboxylase (PYC), malate dehydrogenase (MDH), fumarate hydratase (FH), and succinate dehydrogenase (SDH). The same enzymes were also found in *Lb. gasseri* DSM 20243^T^ genome. All genes are reported in Table 1.

### 2.7. Data Analysis

Analyses were carried out in triplicate and all values were expressed as mean and standard deviation. Two-way Anova tests and t-test analyses (Microsoft Excel for Mac version 11.5) were performed to evaluate significant differences (*p* < 0.05) between means.

## 3. Results

### 3.1. Growth Parameters and Metabolite Production

**Figure 1:**
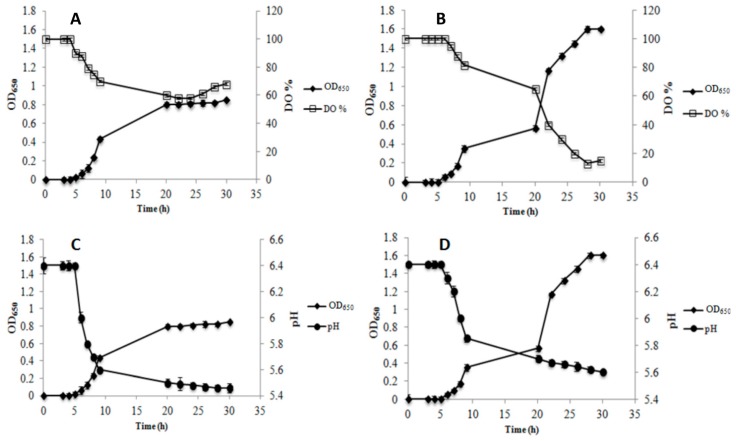
Growth kinetic (OD_650_), dissolved oxygen concentration (DO%) and pH during aerobic (AE, panel **A** and **C**) and respiratory (RS, panel **B** and **D**) cultivation of *Lb. gasseri* AL3 in a batch bioreactor.

**Figure 2:**
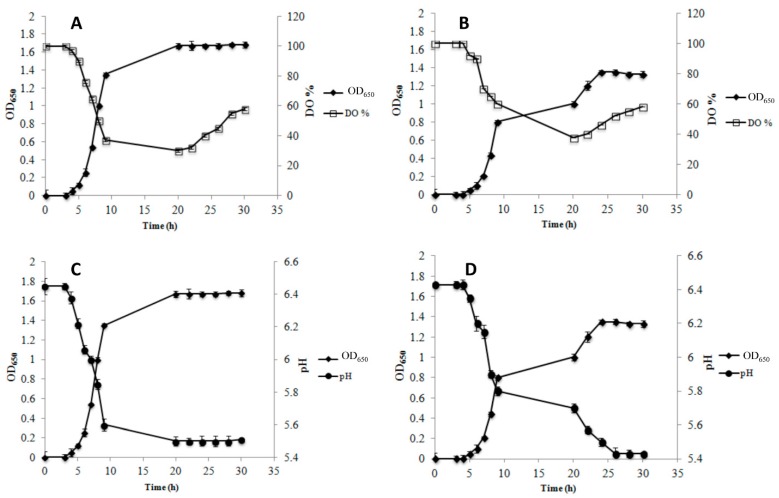
Growth kinetic (OD_650_), dissolved oxygen concentration (DO%) and pH during aerobic (AE, panel **A** and **C**) and respiratory (RS, panel **B** and **D**) cultivation of *Lb. gasseri* AL5 in a batch bioreactor.

The kinetics of growth, DO%, and pH values during AE and RS growth are shown in Figure 1 for strain AL3 and in Figure 2 for strain AL5. Data for AN condition are not reported because neither strain showed the ability to grow when no air or oxygen was injected into the bioreactor.

**Figure 3:**
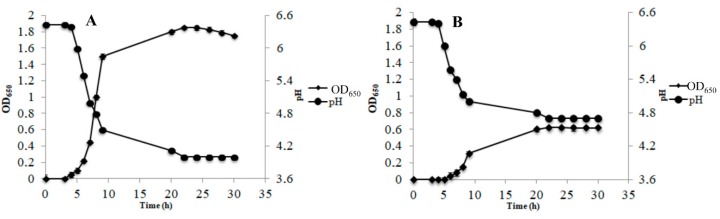
Growth kinetic (OD_650_) and pH during anaerobic (AN, panel **A**) and aerobic (AE, panel **B**) cultivation of *Lb. gasseri* 20243^T^ in a batch bioreactor.

The growth of AL3 was impaired in AE condition compared to in RS condition. In addition, AL3 achieved its highest cell density (OD_650_ = 0.8 ± 0.1) at 20 h of growth in AE conditions (Figure 1A), while in RS condition, AL3 continued to grow up to 28 h, ultimately showing a greater cell density (OD_650_ = 1.65 ± 0.06) (Figure 1B). Of note, the growth curve of AL3 showed a diauxic-like trend in RS condition (Figure 1, panel B). A comparison of the kinetics of DO% showed that AL3 consumed oxygen up to 20 h of growth in AE condition (Figure 1A), but up to 28 h in RS condition (Figure 1B). A pH decrease was observed until the strain entered stationary phase (20 h) during AE growth (Figure 1C), while a very low pH decrease was observed from 20 h onwards in RS condition, despite the increase of cell density until 28 h (Figure 1D). Strain AL5 grew better in AE (Figure 2A) than in RS condition (Figure 2B). The growth of this strain in AE condition was associated with a significant (*p* < 0.05) decrease of both DO% (Figure 2A) and pH (Figure 2C), until the strain entered stationary phase (20 h). On the contrary, growth in RS condition continued from 20 h onwards without oxygen consumption (Figure 2B), but a simultaneous decrease in pH was observed (Figure 2D). Similar to AL3, the growth curve of AL5 showed a diauxic-like trend in RS condition (Figure 2B). As expected, the reference strain *Lb. gasseri* 20243^T^ grew better in AN (Figure 3A) than in AE condition (Figure 3B) and was not able to grow in RS condition.

*Lb. gasseri* 20243^T^ achieved the highest cell density (OD_650_ = 1.8 ± 0.09) after 22 h and dramatically reduced the pH of the medium. AE condition heavily impaired the growth of *Lb. gasseri* 20243^T^ (Figure 3B) and the strain was not able to consume oxygen.

**Figure 4:**
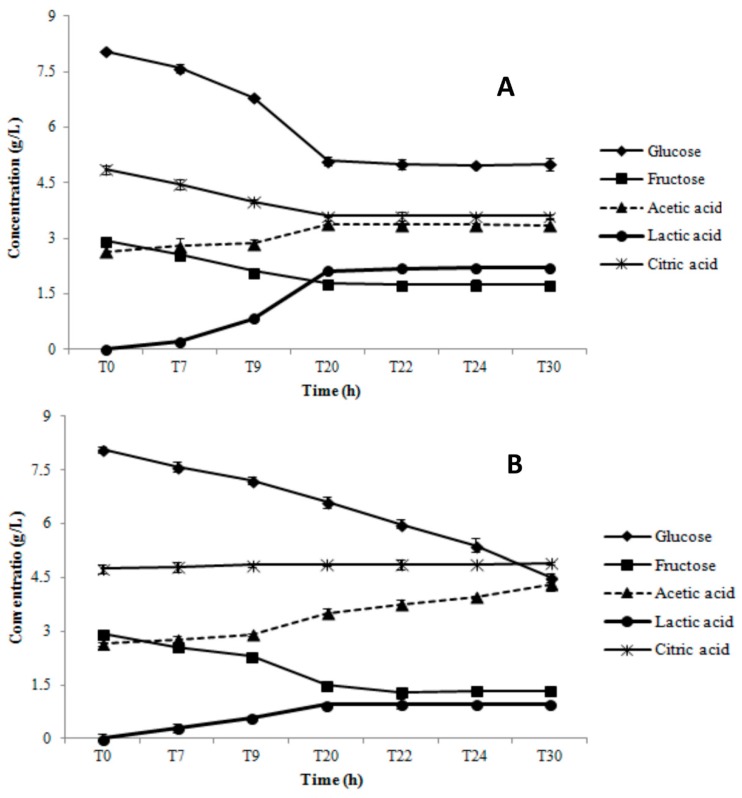
Substrate consumption and metabolites production during aerobic (AE, panel **A**) and respiratory (RS, panel **B**) cultivation of *Lb. gasseri* AL3 in a batch bioreactor.

**Figure 5:**
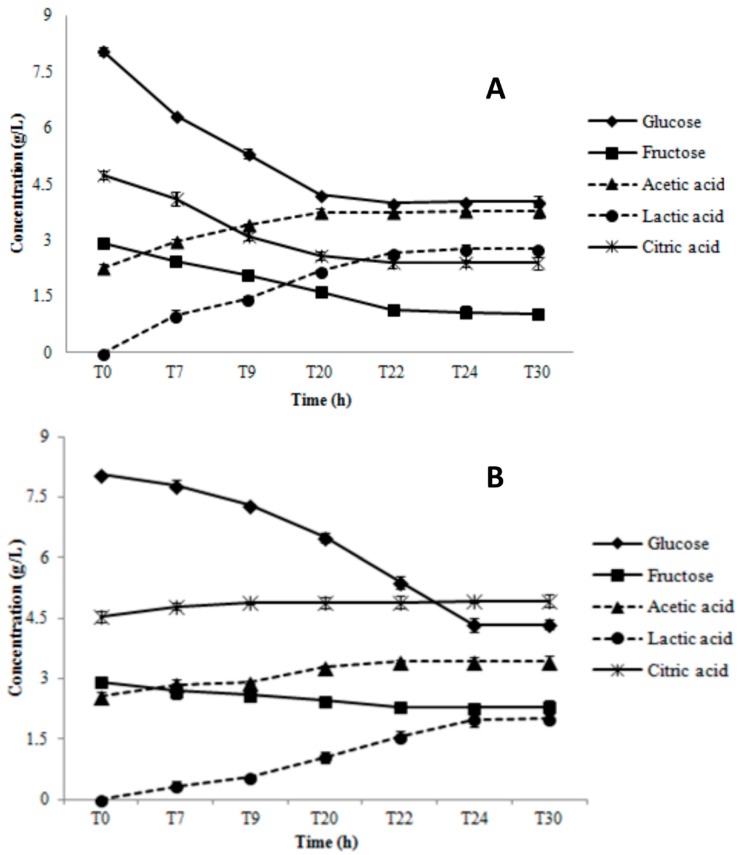
Substrate consumption and metabolites production during aerobic (AE, panel **A**) and respiratory (RS, panel **B**) cultivation of *Lb. gasseri* AL5 in a batch bioreactor.

Results of the substrate consumption and metabolite production tests during growth in AE and RS conditions are shown in Figure 4 for AL3 and Figure 5 for AL5.

**Figure 6:**
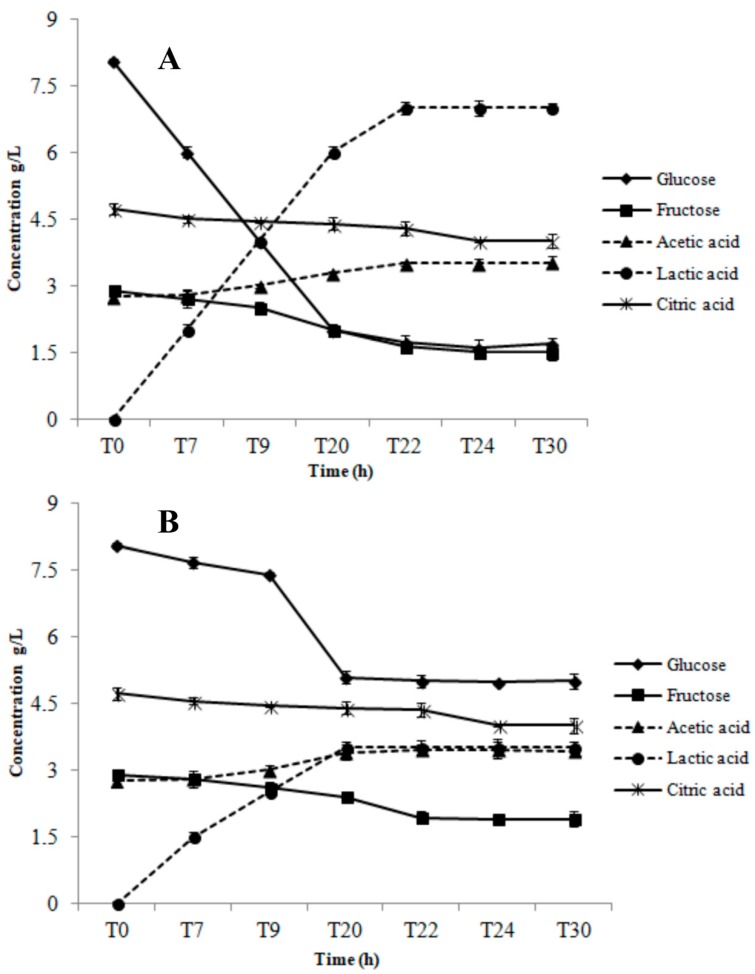
Substrates consumption and metabolites production during anaerobic (AN, panel **A**) and aerobic (AE, panel **B**) cultivation of *Lb. gasseri* 20243^T^ in a batch bioreactor.

AL3 consumed glucose (2.8 ± 0.1 g/L), fructose (1.0 ± 0.08 g/L), and citric acid (0.7 ± 0.09 g/L) only throughout the first 20 h of growth in AE (Figure 4A). Lactic acid was the main end-product (2.45 ± 0.1 g/L), but a small quantity of acetic acid (0.35 ± 0.1 g/L) was also produced (Figure 4A). In RS condition, there was constant glucose consumption during the whole monitoring period (3.6 ± 0.1 g/L), which was remarkably higher than in AE condition (Figure 4B). However, the trend and quantity of fructose depletion (1.1 ± 0.09 g/L) in RS was similar to that in AE condition (Figure 4B). The highest lactic acid concentration (1.02 ± 0.06 g/L) was obtained at 20 h, significantly lower than that registered in AE condition (2.45 ± 0.1 g/L). However, we also observed an acetic acid production level of up to 1.9 ± 0.05 g/L at 30 h. AL5 consumed glucose (3.7 ± 0.08 g/L), fructose (1.56 ± 0.1 g/L), and citric acid (1.6 ± 0.07 g/L) until 22 h of growth in AE condition (Figure 5A). Moreover, a significant amount of acetic (1.5 ± 0.08 g/L) and lactic acid (2.6 ± 0.04 g/L) was produced (Figure 5A). The same glucose consumption (3.75 ± 0.1 g/L) but a lower acetic (0.65 ± 0.05 g/L) and lactic (2.1 ± 0.06 g/L) acid production were observed in RS condition (Figure 5B). No significant (*p* > 0.05) consumption of fructose and citric acid was registered. As shown in Figure 6, *Lb. gasseri* 20243^T^ was able to consume only glucose and to produce only lactic acid in both AN (Figure 6A) and AE condition (Figure 6B). In AN condition, *Lb. gasseri* 20243^T^ consumed glucose (6.3 ± 0.08 g/L) until 22 h and produced lactic acid as the main end-product (7.1 ± 0.09 g/L) (Figure 6A). In AE condition (Figure 6B), the highest glucose depletion (3.40 ± 0.06 g/L) was registered at 20 h and the acetic acid production (3.05 ± 0.07 g/L) was significantly lower than that registered in AN condition.

### 3.2. Survival of the Cells Under Cold-Starvation Stress

**Figure 7:**
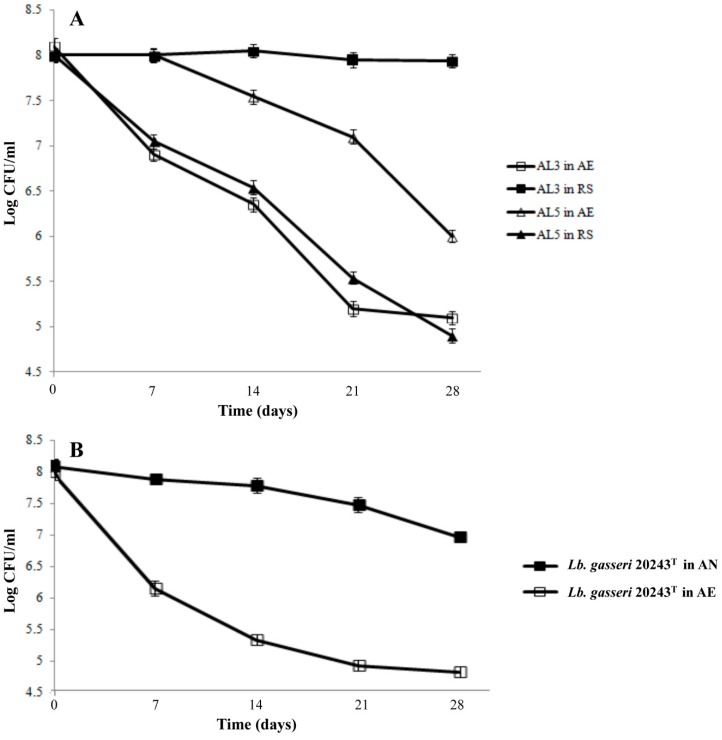
Viable counts (Log CFU/mL) of *Lb. gasseri* AL3 and AL5 strains cultivated under aerobic (AE) and respiration (RS) conditions (**A**) and viable counts (Log CFU/mL) of *Lb. gasseri* DSM 20243^T^ cultivated under anaerobic (AN) and aerobic (AE) conditions (**B**) along 28 days of starvation at 4 °C.

Results of viable counts after 28 days of starvation at 4 °C are shown in Figure 7A.

Only the cells of AL3 from RS condition exhibited tolerance under cold-starvation stress. Indeed, no significant (*p* > 0.05) difference in cell load between starting population and that after 28 days under stress was registered. However, the AE cultivation strongly impaired the viability of both strains. In particular, at the end of the storage, a significant (*p* < 0.05) reduction of 3.08 Log and 2.0 Log cycles was observed for aerobic AL3 and AL5 cultures, respectively. As shown in Figure 7B, the *Lb. gasseri* 20243^T^ anaerobic culture exhibited the highest tolerance to cold-starvation stress.

### 3.3. Comparative in Silico Analysis

Results of the comparative in silico analysis conducted on 33 genes are summarized in Table 1. The strains AL3 and AL5 show the same profile, apart the gene encoding for the flavoprotein L-amino acid oxidase (*lao*, in the group of aerobic metabolism genes) that is present only in the AL3 genome. All the genes involved in the aerobic pathway that we analysed are present in AL3 and AL5 genome, wih exclusion of L-amino acid oxidase in AL5. Instead, genome of *Lb. gasseri* 20243^T^ is lacking of the *lox* (lactate oxidase) and *nox* (NADH oxidase) genes. All the genes of respirartory metabolism investigated in this study (*ndh*, *ubiE* and *cydABCD*) are present in all the three strains. Of the 13 stress response genes taken into account in this work, only 7 were present in the genome of both strains AL3 and AL5, which interestingly have the gene for the synthesis of superoxide dismutase (*SOD*). On the other hand, *Lb. gasseri* 20243^T^ is lacking *GshA* (γ-glutamylcystiene synthetase) and *SOD* (superoxide dismutase) compared to AL3 and AL5. Remarkably, we found in the genome of AL3 and AL5 most the genes encoding for the enzymes involved in the citrate metabolism. Results of comparative analysis showed that the type strain *Lb. gasseri* 20243^T^ is lacking *CitD*, *CitE*, *CitF, CitP*, *MDH* and *FH* genes.

## 4. Discussion

In this work, for the first time, the ability of *Lactobacillus gasseri* strains to shift towards aerobic and/or respiratory metabolism was investigated during growth in a bioreactor and the effect of different conditions (AE, RS, AN) on growth performance and starvation stress tolerance was evaluated. Moreover, the metabolic profiles of AL3 and AL5 strains were investigated to provide additional biochemical evidence of the possible activation of aerobic and/or respiratory pathway. The type strain *Lb. gasseri* DSM 20243^T^ was included in this study for a comparative purpose. Our results showed that *Lb. gasseri* AL3 grew better in RS while AL5 grew better in AE. Surprisingly, both strains didn’t show any growth in AN. On the other hand, the strain *Lb. gasseri* 20243^T^ grew better in AN condition, and the presence of oxygen and respiratory cofactors (heme and menaquinone) negatively affected its growth performance. The inability of AL3 and AL5 to growth in AN could be imputable to an inadequate ATP generation. According to Hertzberger et al. [16] the growth of some *Lactobacillus* could be inhibited by the absence of C1 and C2 compounds like CO_2_ and acetate, respectively. They are produced by POX and ACK from pyruvate only in presence of oxygen. When we inject nitrogen into the substrate a gas stripping could occur, with a consequent CO_2_ depletion. However, this cannot explain the different behaviour in AN condition between AL3 and AL5 and *Lb. gasseri* 20243^T^. Even though *Lb. gasseri* is a microorganism described as an inhabitant of intestine, despite the predominantly anaerobic environment of gut, close to the mucosal tissues an oxygen gradient may be encountered [16]. Difference in growth kinetics and metabolic profiles of the three strains clearly suggests that several metabolic changes underlie the physiological characteristics observed. Moreover, results of comparative in silico analysis revealed differences in the genomic profile of *Lb. gasseri* 20243^T^ compared to that of AL3 and AL5 strain. In RS cultivation, AL3 showed increased biomass production, reduced acidification, and higher oxygen consumption. These results suggest the activation of a respiratory metabolism. Comparing the kinetics of growth, substrate consumption, and metabolite production, we hypothesize a double metabolism for this strain: it may first grow via fermentation and then via respiration. In the first 20 h, glucose is metabolized to produce mainly lactic acid, and this could reasonably explain the observed pH decline. After this time, the glucose consumption continued but only acetic acid was produced. In fact, a very low decrease in pH was observed, despite AL3 continuing to grow and to consume oxygen. Similar results were found for a strain of *Lactococcus lactis* subsp. *lactis* by Duwat et al. [20]. These authors found that during the respiratory cultivation, obtained in presence of oxygen, hemin and menaquinone, microorganism shifted towards a respiratory metabolism. In particular, glucose consumption and lactic acid accumulation was registered in the first 7 h of growth, then glucose consumption continued but a reduction in lactic acid accumulation was observed, accompanied by an increase of acetate concentration. The hypothesis of a double metabolism could also explain the diauxic-like growth pattern observed. The activation of the electron transport chain could explain the high oxygen consumption (by cytochrome oxidase action) and the increased cell density (because of extra ATP generation) observed after 20 h of growth. Moreover, the production of acetate instead of lactate is one of the typical metabolic changes associated with respiratory growth [20,21,22]. AL3 possesses the main genes involved in respiratory metabolism, including NADH dehydrogenase, the cytochrome oxidase operon, and ubiquinone/menaquinone biosynthesis methyltransferase [19]. The ability of AL3 to shift toward a respiratory metabolism has also probably contributed to a remarkable improvement in cell survival under starvation stress. The analysis of the AL3 genome sequence [19] revealed a large pattern of genes involved in oxidative stress resistance mechanisms, including superoxide dismutase, NADH peroxidase, complete thioredoxin-thioredoxin reductase system, as well as member of the DNA binding proteins from starved cells which are able to provide cell protection during exposure to environmental stress, including nutritional deprivation. The robustness to starvation stress in long term survival was also previously demonstrated in respiration-competent strains of *Lb. plantarum* [10], *Lb. casei* [4], and *Lactococcus lactis* [23,24].

Strain AL5 showed the best growth performance in AE condition. According to the results of the AL5 draft genome analysis [19], AL5 possesses genes predicted to encode for both POX and acetate kinase (ACK), the main enzymes involved in the aerobic pathway. Therefore, the POX-ACK pathway activation in the presence of oxygen could explain the production of acetate and the increased biomass production, the former probably because of pyruvate conversion and the latter because of the generation of an extra ATP. Of note, both strains metabolized citric acid in AE condition, although AL5 did so more than AL3. The draft genome of both strains showed the sequence encoding for CitP, a transporter belonging to the 2-hydroxycarboxylate transporter family which is involved in uptake system of citrate [25,26]. Interestingly, our results showed citrate depletion in the first growth period, even though the pH did not decrease rapidly. Moreover, we found sequences encoding for all subunits (gamma, beta, and alpha) of CL, the enzyme involved in citrate metabolism. In fact, this enzyme catalyzes the cleavage of citrate into oxaloacetate and acetate [25,27,28]. While the sequence encoding for the AOD enzyme, responsible of oxaloacetate conversion into pyruvate, was not found, BLAST analysis revealed the presence of the reductive TCA cycle genes encoding for MDH, FH, and SDH enzymes. Lactobacilli usually lack a complete TCA cycle, even though some strains are able to metabolize citrate via the reductive TCA cycle or via pyruvate lyase-oxaloacetate decarboxylase pathway [1,25]. Accordingly, Kang et al. [25] identified MDH, FH, and SDH as the enzymes involved in citrate conversion into succinate, via the reductive TCA cycle, in *Lactobacillus panis* PM1. To the best of our knowledge, this is the first time that sequences encoding for CitP enzyme has been annotated in the *Lb. gasseri* genome, while, sequences encoding for CL were annotated only in *Lb. gasseri* 32 (IMG/M database, https://img.jgi.doe.gov). As a matter of fact, CL and CitP occurrence is very limited among Lactobacilli genomes [1,25,29]. Although AE was the best growth condition for AL5, the strain showed a very low resistance to starvation stress. It has been demonstrated that the activation of the aerobic pathway, and/or of other enzymes involved in oxygen utilization, may result in a high production of H_2_O_2_ [30]. Hertzberger et al. [16] found that the endogenous production of H_2_O_2_ is the main cause of oxidative stress in the probiotic *Lb. johnsonii* NCC 533 during aerobic growth. In the draft genome of AL5 we did not find sequences encoding for the catalase or pseudocatalase enzymes. Therefore, the possible toxic effect of H_2_O_2_ accumulation can explain the decreased survival of AL5 during long-term storage. Although the unique difference between AL3 and AL5 is in the presence of *lao* gene encoding for the flavoprotein L-amino acid oxidase, the two strains clearly showed a different energetic metabolism. All the genes here taken into account are that responsible of energetic metabolism, then we can suppose that difference is due to a different expression level of these genes. Regarding to *Lb. gasseri* 20243^T^, it showed the typical homo-fermentative behaviour. As expected, the anaerobiosis was the better growth condition, the glucose was the preferred carbon source and lactic acid was the main fermentation end-product. On the other hand, result of comparative in silico analysis revealed that *Lb. gasseri* 20243^T^ genome lacks genes encoding for NOX, LOX, L-amino acid oxidase, SOD and GshA enzymes. It was been noted that the mentioned enzymes are involved in oxygen tolerance and oxidative stress protection mechanisms in LAB [1]. Therefore, these evidences could explain the poor adaptation ability of *Lb. gasseri* 20243^T^ to AE condition. Moreover, *Lb. gasseri* 20243^T^ genome lacks genes encoding for CitP, SDH and all subunits of CL enzyme, therefore its inability to use citrate is not surprising. On the contrary, despite the presence of genes encoding for components of a minimal respiratory chain of LAB (NDH, UbiE and CydABCD), *Lb. gasseri* 20243^T^ was not able to grow in RS condition.

In conclusion, this work contributes to the understanding of the adaptation response of *Lb. gasseri* strains to respiratory and aerobic metabolism. To date, this is the first study in which aerobic and respiratory growth has been evaluated in *Lb. gasseri* strains in batch cultivation. *Lb. gasseri* AL3 is confirmed to have a respiratory phenotype and AL5 an aerobic phenotype. We found that neither strain grew under anaerobiosis, the typical growth condition of *Lb. gasseri*. The differences in growth kinetics and in metabolite profiles clearly suggest that several metabolic changes underlie the physiological characteristics observed. We demonstrated that a potential activation of respiratory metabolism can provide several advantages, such as improved biomass production and robustness during long-term storage. These features may be relevant in several technological and health-promoting applications, including starter and/or probiotic formulations. Further investigations will be performed on gene expression in AL3 and AL5, using a transcriptomic and proteomic approach to confirm the results of this study.

## Figures and Tables

**Table 1:** Results of in silico analysis of genes (presence [+]/absence [−]) involved in aerobic and respiratory metabolism, stress response and in the partial tricarboxylic acid (TCA) cycle in *Lb. gasseri* AL3, AL5 and DSM 20243^T^ genomes from NCBI database (http://www.ncbi.nlm.nih.gov).

Group	Genes	Strains		
		AL3	AL5	DSM 20243^T^
Aerobic metabolism	*pox* (pyruvate oxidase)	+	+	+
	*ack* (acetate kinase)	+	+	+
	*lox* (lactate oxidase)	+	+	−
	*nox* (NADH oxidase)	+	+	−
	*lao* (L-amino acid oxidase)	+	−	−
Respiratory metabolism	*ndh* (NADH dehydrogenase)	+	+	+
	*ubiE* (ubiquinone/menaquinone biosynthesis methyltrasferase)	+	+	+
	*cydABCD* (cytochrome bd-I oxidase operon)	+	+	+
Stress response	*npr* (NADH peroxidase)	+	+	+
	*gor* (glutathione reductase)	+	+	+
	*gop* (glutathione peroxidase)	−	−	−
	*GshA* (γ-glutamylcystiene synthetase)	+	+	−
	*GshB* (glutathione synthetase)	−	−	−
	*GshF* (bifunctional glutamate-cysteine ligase/glutathione synthetase)	−	−	−
	*TrxR* (thioredoxin reductase)	+	+	+
	*TrxP* (thioredoxin peroxidase)	+	+	+
	*SOD* (superoxide dismutase)	+	+	−
	*Kat* (catalase)	−	−	−
	*MnKat* (Manganese-catalase)	−	−	−
	*KatG* (catalase-peroxidase)	−	−	−
	*Dps* (DNA binding protein from starved cells)	+	+	+
Partial tricarboxylic acid (TCA) cycle	*CitD* (gamma subunits of citrate lyase)	+	+	−
	*CitE* (beta subunits of citrate lyase)	+	+	−
	*CitF* (alpha subunits of citrate lyase)	+	+	−
	*CitP* (citrate permease)	+	+	−
	*AOD* (oxaloacetate decarboxylase)	−	−	−
	*PYC* (pyruvate carboxylase)	−	−	−
	*MDH* (malate dehydrogenase)	+	+	+
	*FH* (fumarate hydratase)	+	+	+
	*SDH* (succinate dehydrogenase)	+	+	−
